# A Novel Mixed Perovskite-like Alkali Niobium Tungsten Oxide (Na_*x*_(Nb_0.5_W_0.5_)O_3_) Synthesized by a Sustainable Collagen Dispersion Process

**DOI:** 10.1021/acsomega.6c02992

**Published:** 2026-09-21

**Authors:** Gabriel Luiz Rasch, Adilar Gonçalves dos Santos Júnior, Ricardo Faccio, Fabiano Bernardi, Andrés Cuña Suárez, Antonio Marcos Helgueira de Andrade, Leonardo Moreira dos Santos, Roberto Hübler, Wladimir Hernandez Flores, Célia de Fraga Malfatti

**Affiliations:** † Corrosion Research Laboratory (LAPEC), Engineering School, 28124 Federal University of Rio Grande do Sul (UFRGS), Porto Alegre, Rio Grande do Sul 90040-060, Brazil; ‡ Área Física & Centro Nanomat, DETEMA, Facultad de Química, 201894 Universidad de la República, Av. Grail. Flores 2124, C.P., 11800 Montevideo, Uruguay; § Institute of Physics, Federal University of Rio Grande do Sul (UFRGS), Porto Alegre, Rio Grande do Sul 90040-060, Brazil; ∥ Area Fisicoquímica, DETEMA, Facultad de Química, Universidad de la República, 11800 Montevideo, Uruguay; ⊥ Laboratorio de Energías Renovables, IPTP, Facultad de Química, Universidad de la República, Pando, Canelones 91000, Uruguay; # Materials and Nanoscience Laboratory, Polytechnic School, Pontifical Catholic University of Rio Grande do Sul, Porto Alegre 90619-900, Rio Grande do Sul, Brazil; ∇ Federal University of Pampa, Bagé Campus, Avenue Maria Anunciação Gomes de Godoy 1650, 96400-100 Bagé, RS, Brazil

## Abstract

Niobium compounds show considerable structural diversity and can be classified as binary compounds, alkali niobates, or complexes. Although niobium oxides have shown promising results as battery anodes, the perovskite structures are rarely utilized for electrochemical energy storage applications due to their low electrical conductivity. Therefore, this study reports the synthesis of a novel mixed perovskite, Na_*x*_(Nb_0.5_W_0.5_)­O_3_, through a green, collagen-assisted route. FTIR analysis indicated that the synthesis is facilitated by the possible complexation of the niobium precursor within the protein structures. Furthermore, Rietveld refinement confirmed the formation of a mixed compound consisting of 97% Na_*x*_(Nb_0.5_W_0.5_)­O_3_ and 3% (Na_0.75_Nb_0.25_)­WO_4_. FEG-SEM characterization revealed nanoparticles with an average minimum Feret diameter of 95.03 nm. Postactivation XPS analysis of the electrodes revealed that the charge storage occurs via intercalation-type pseudocapacitance. Another significant aspect is the stabilization of tungsten in the +6 oxidation state, leading to an enhanced theoretical maximum capacity of 270.9 mAh g^–1^. Finally, galvanostatic charge and discharge confirmed the energy storage process, demonstrating a capacity of 243 mAh g^–1^ at 200 mA g^–1^ in an aqueous sodium-ion electrolyte. This enhanced performance is likely attributed to improved material conductivity resulting from the reduction of the bandgap from 3.4 eV (NaNbO_3_) to 3.04 eV and an *R*_ct_ of 0.48 Ω by electrochemical impedance spectroscopy.

## Graphical abstract

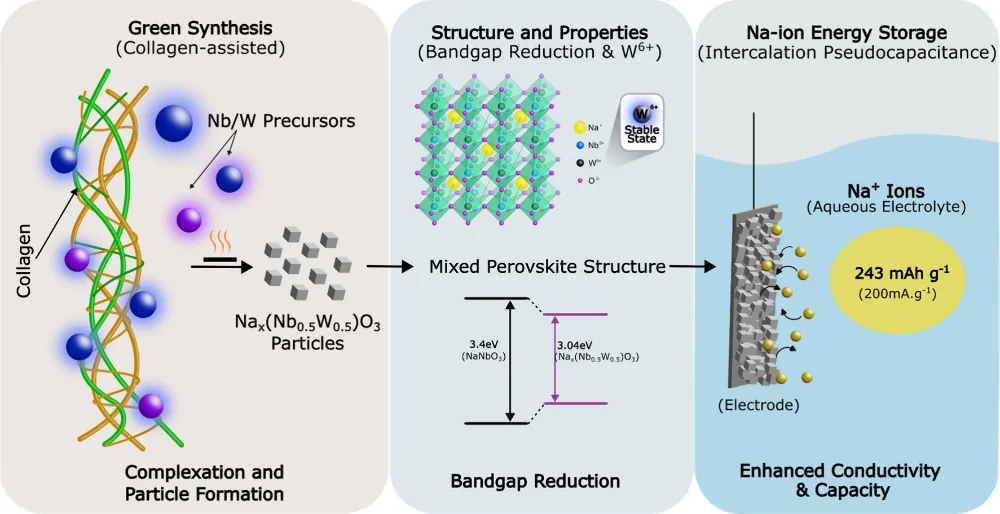

## 1 Introduction

The structural chemistry of niobium oxides is remarkably rich and diverse. The first group, characterized by stoichiometric simplicity, comprises binary compounds such as Nb_2_O_5_ and NbO_2_, as well as substoichiometric compositions like Nb_2_O_5–*x*_. Nb_2_O_5_ itself can exhibit distinct crystalline arrangements, including pseudohexagonal, orthorhombic, and monoclinic phases.^1,2^ Another class comprises alkali niobates,^1,1,3–5^ such as NaNbO_3_, KNbO_3_, and LiNbO_3_, which exhibit diverse crystalline arrangements, most commonly associated with perovskite-type structures, although more complex structures may also be obtained. In this context, heteroatom insertion and defect engineering have been active areas of research, as these modifications tend to broaden the material’s scope of applications.^6^

One of the areas most impacted by such discoveries is energy storage.^2,7–11^ Many studies have reported the application of binary niobium oxide or phosphates compounds as possible anodes for fast-charging lithium-ion batteries^10–13^ and, in some cases, sodium-ion systems.^1,14–18^ Regarding perovskite structures, specifically NaNbO_3_, the majority of publications have focused on properties such as piezoelectricity,^1,19–21^ antiferroelectricity,^1,21^ and applications in sensors.^22^ In addition, its semiconductivity, with a bandgap of *E*_b_ = 3.4 eV,^1^ enables applications in photocatalysis.^23–25^ There are also reports on lead-free dielectric capacitors^1,26^ as sustainable alternatives. However, given its low electrical conductivity, it is rarely reported in electrochemical energy storage applications.^1,1^ In the context of mixed oxides, studies focused on niobium–tungsten solid solution have shown promising results. Structural modifications, such as the insertion of ions with different valences, have improved the conductivity of the material.^1,27,28^ Furthermore, nanoengineering of the material affects ion diffusion within the structure by reducing the insertion path, which tends to improve the efficiency of the intercalation process.^1^

While the integration of tungsten ions in the crystal lattice presents its own challenges, material nanostructuring typically relies on techniques requiring high pressures^29–31^ and advanced processes,^23,32^ usually demanding the use of nonrenewable reagents and solvents.^33^ In the context of climate change, it is essential to develop new technologies that prioritize sustainability from their inception. A previous study addressing the genotoxicity of Nb_2_O_5_ nanoparticles employed a green synthesis route utilizing collagen as a structural matrix;^34^ however, the intrinsic mechanisms of this process remained unexplored. Furthermore, the use of ammonium niobium oxalate (ANO) as a precursor hinders the synthesis of mixed oxides, as the majority of transition metals tend to form insoluble complexes with the oxalate anion.^35^

Addressing these limitations, the present study reports the synthesis of a novel nanostructured niobium-based complex oxide, highly substituted with tungsten (perovskite Na_*x*_(Nb_0.5_W0_0.5_)­O_3_), obtained via a green synthesis route. Additionally, ammonium niobium oxalate (NH_4_[NbO­(C_2_O_4_)_2_(H_2_O)_2_]·*n*H_2_O, ANO) was used as a precursor in an aqueous medium with collagen as a structural matrix for nanostructure formation. This material was characterized in terms of morphological and structural properties using FTIR, XRD, FEG-SEM, and XPS, electronic properties by UV–vis spectroscopy, and electrochemical properties by cyclic voltammetry (CV), galvanostatic charge–discharge (GCD), and electrochemical impedance spectroscopy (EIS).

## 2 Materials and Methods

### 2.1 Synthesis of Mixed Nb/W Perovskite (NWO)

The synthesis of the materials was carried out via a collagen dispersion process. For the synthesis of NWO, 4.41 g of collagen was dissolved in 35 mL of ultrapure Milli-Q water at 45 °C for 1 h under magnetic stirring. Simultaneously, 1 g of purified ammonium niobium oxalate (NH_4_[NbO­(C_2_O_4_)_2_(H_2_O)_2_]·*n*H_2_O, CBMM) was dissolved along with 0.7661 g of Na_2_WO_4_·2H_2_O (Reagen) in 35 mL of Milli-Q water. The solution was kept under magnetic stirring for 1 h without heating. Subsequently, the solution containing the metal precursors was added dropwise to the collagen solution, which was kept under constant magnetic stirring. The resulting solution was stirred for 20 min at 45 °C. Finally, the mixture was transferred to a 150 mL crucible and kept at 80 °C for 48 h in a drying oven open to the atmosphere. Subsequently, the crucible containing the resin was transferred to a muffle furnace open to the atmosphere, where it was carbonized at 250 °C for 3 h at a heating rate of 5 °C min^–1^ and then allowed to cool naturally to room temperature. Afterward, the formed carbon foam was ground using a mortar and pestle and transferred back to the muffle furnace, where it was calcined at 700 °C for 3 h at a heating rate of 5 °C min^–1^ and allowed to cool naturally. The resulting material was then dispersed in 35 mL of Milli-Q water, placed in a Falcon tube, and sonicated using a probe sonicator at 850 W for 10 min. The dispersion was then centrifuged at 4000 rpm for 20 min. Afterward, the supernatant was discarded, and another 35 mL of Milli-Q water was added. This procedure was repeated three times. The precipitate was transferred to a crucible and dried in an oven open to the atmosphere at 80 °C for 24 h. A summary of the synthesis process is shown in Figure 1. For comparison, samples of niobium pentoxide (Nb_2_O_5_) were prepared using the same method. The ammonium niobium oxalate purification process is available in the Supporting Information (SI) (Section S1), and the thermal analysis of the carbonized NWO/collagen precursor is available in the Supporting Information (Section S2).

**Figure 1:**
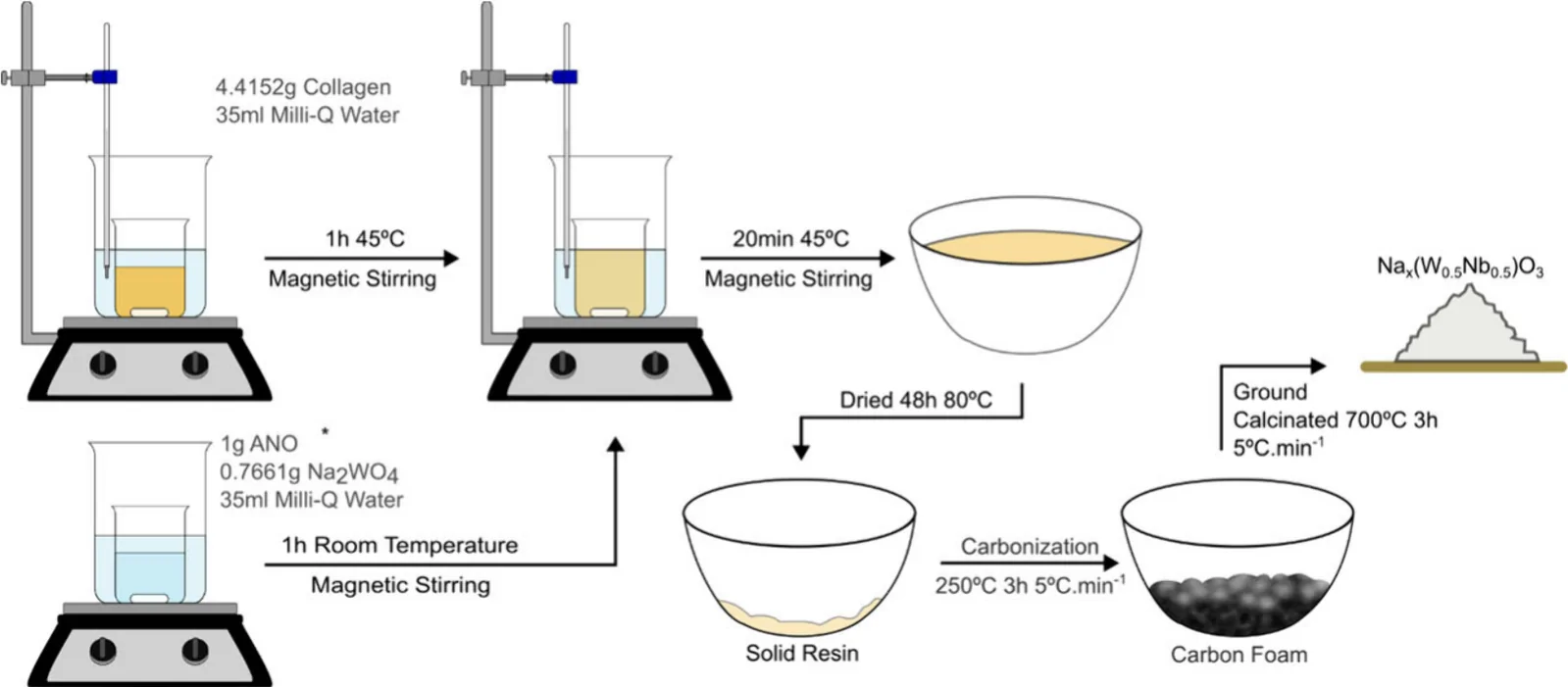
Schematic representation of the Na_*x*_(Nb_0.5_W_0.5_)­O_3_ synthesis process.

### 2.2 Material Characterization

For the evaluation of the chemical properties related to material synthesis, a comparative study between the synthesis containing only the niobium precursor and that containing both metals (Nb and W) was performed. Samples obtained after the carbonization step and samples of the final materials were pelletized with KBr and analyzed in an FTIR spectrometer Vertex 70 V (Bruker) with an RT-DLaTGS detector, operating in transmittance mode. Data acquisition was done using OPUS software (Bruker) in the range of 400 to 4000 cm^–1^, with 500 scans and a resolution of 4 cm^–1^. The conversion of the transmission data to absorbance was performed with the same software.

Regarding the crystalline arrangement, the analysis was conducted in a Rigaku MiniFlex 600-C X-ray diffractometer, operating in Bragg–Brentano θ–2θ geometry. The equipment used Cu Kα radiation (λ = 1.5419 Å), operating at 40 kV and 15 mA, and a D/teX Ultra2 1D detector. The diffraction pattern was obtained in the 2θ scan mode from 3° to 90°, with a step size of 0.01° and a scan rate of 0.5° min^–1^. Structural and microstructure parameters were obtained by Rietveld refinement, using the GSAS-II software.^36,37^ The refinement was carried out over the 10–90° 2θ range. The A-site occupancy of the minority scheelite-type phase was fixed by charge neutrality with W­(VI) and was not refined, since the site occupancy and displacement parameter of that site are strongly correlated for a 3 wt % phase in laboratory Cu Kα data; the Nb:W ratio of the main phase was likewise fixed at its nominal value.

The morphological analysis of the material was performed using an FEG-SEM ZEISS EVO LS-10 instrument, operating at 20 kV. The particle size distribution was determined by measuring the maximum and minimum Feret diameters^38^ using the ImageJ 1.54g software.

XPS measurements were performed to investigate the chemical components present at the sample surface. For the measurements, a thin layer of the powder was dispersed on copper tape (NWO sample) or directly placed onto a molybdenum sample holder (NWO electrode) and exposed to the ultrahigh vacuum (UHV) conditions inside the analysis chamber. The base pressure during the measurements was approximately 10^–9^ mbar. The XPS measurements were performed at room temperature utilizing an Al Kα X-ray source (1486.6 eV). Spectra were collected in the and high-resolution regions corresponding to Nb 3d, W 4f, Na 1s, O 1s, and C 1s.

The electron analyzer (Omicron Sphera) was set at a pass energy of 50 and 10 eV, with an energy step of 1.0 and 0.1 eV, and an acquisition time of 0.2 and 2 s/point for the survey and high-resolution scans, respectively. Charging effects were corrected by referencing the adventitious carbon component to 284.5 eV. The XPS spectra were adjusted with XPSPeak 4.1 software. A Shirley-type background was applied, and symmetric Gaussian–Lorentzian sum functions with an 18% Lorentzian contribution were used to fit the spectra. The Lorentzian contribution was determined from the Au 4f XPS spectrum of a gold standard foil. The relative binding energy and full width at half-maximum (fwhm) value of a given component were constrained to identical values across the different samples analyzed.

The material bandgap was determined by UV–vis diffuse reflectance spectroscopy using a Cary 5000 spectrophotometer (Agilent) in the wavelength range of 200–800 nm. The data were treated using the Tauc method,^39^ considering NWO as a material with an indirect electronic transition, since sodium niobate has this property.^40^ Initially, the value of the Kubelka–Munk function, *F*(*R*), for each point was obtained by eq 1,^41^ where *R* is the measured reflectance. Subsequently, the photon energy (*h*ν) was plotted against (*F*(*R*)*h*ν)^1/2^, and a linear regression was performed using eq 2 ^42^ in the inclined linear region. The optical bandgap energy (*E*_b_) was determined from the intercept where (*F*(*R*)*h*ν)^1/2^ = 0.

1 $$F\left(R\right)=\frac{{\left(1-R\right)}^{2}}{2R}$$

2 $${\left(F\left(R\right)h\nu \right)}^{1/2}=B\left(h\nu -E_{b}\right)$$

where *R* is the reflectance, *h* is Planck’s constant, ν is the electromagnetic wave frequency, and *E*_b_ is the bandgap energy.

### 2.3 Electrochemical Characterization

For the electrochemical study of the electrodes produced with the new material, two distinct fabrication routes were employed. This was necessary to avoid overlap between the oxygen peaks of polyvinyl acetate (PVAc) and NWO during the X-ray photoelectron spectroscopy (XPS) analysis, which required the use of a fluorinated polymer (polyvinylidene fluoride, PVDF).

For the activation mechanism study, working electrodes were prepared by mixing NWO, carbon black (Carbon Vulcan XC72R, Cabot), and polyvinylidene fluoride (PVDF, Sigma-Aldrich) in a weight ratio of 80:10:10 (wt %), respectively, with a solids content of 20 wt % in *N*-methyl-2-pyrrolidone (NMP, Sigma-Aldrich). Initially, NWO, carbon black, and NMP were stirred at 5000 rpm using a high-shear mixer (IKA T18 Ultra-Turrax) for 10 min. Subsequently, a 10 wt % PVDF solution in NMP was added to the system, which was then stirred at 5000 rpm for 1 h.

The resulting slurry was drop-cast onto a flexible graphite sheet (GFS) previously abraded with sandpaper and washed sequentially with acetone, ethanol, and water. The electrodes were then dried in a conventional oven at 100 °C overnight, resulting in a mass loading of 2–3 mg cm^–2^. Finally, the electrodes were pressed using a hydraulic press at 1 MPa, and an active area of 0.5 cm^2^ was delimited using 3M 470 tape.

Electrode activation was carried out in a three-electrode cell, using Hg/HgO 1 M KOH as the reference electrode (RE) and a platinum plate as the counter electrode (CE). An aqueous solution of 1 M NaOH was employed as the electrolyte. The process consisted of the use of 13 consecutive cyclic voltammetry (CV) scans performed at a scan rate of 50 mV s^–1^, in the potential window from +0.6 to −1.1 V vs Hg/HgO, using an Autolab PGSTAT302N potentiostat/galvanostat with data acquisition via NOVA 2.1.4 software (Metrohm, Herisau, Switzerland).

The comparison before and after activation was conducted in a three-electrode cell using saturated Ag/AgCl as the RE and a platinum plate as the CE, in an electrolyte composed of 1 M Na_2_SO_4_ (Merck) + 0.8 mM sodium dodecyl sulfate (SDS, Synth). To overcome the highly hydrophobic nature of the PVDF binder, we incorporated SDS into the system to improve the wettability of the electrode surface. Measurements were performed using a PAR273A potentiostat/galvanostat (Princeton Instruments) with data acquisition by Powersuite 2.58 software (Princeton Instruments).

Following the activation study, new electrodes were produced using a different fabrication route. For these electrodes, the slurry was prepared using an 80:15:5 mass ratio of NWO, Vulcan XC72R (Cabot), and polyvinyl acetate (PVAc, Denver Química) with a solid content of 25%. Initially, an ethanol–water solution (30% v/v EtOH in Milli-Q water) was prepared, into which the active material and the conductive additive were added and stirred using a high-shear mixer (IKA T18 Ultra-Turrax) at 7000 rpm for 15 min. Subsequently, a 7.5% PVAc ethanolic solution was added to the system, which was left under stirring at 7000 rpm for an additional 15 min. The resulting slurry was deposited by tape casting onto flexible graphite current collectors, which were previously prepared as described in the previous procedure. The electrode was allowed to dry for 24 h in an oven at 100 °C and then calendered. The active area of the electrode was delimited using 3M 470 tape to an area of 0.5 cm^2^, with a mass loading of 1.5 to 2 mg cm^–2^.

All electrochemical tests with these electrodes used a 1 M Na_2_SO_4_ electrolyte, a saturated Ag/AgCl reference electrode, and a graphite counter electrode in a three-electrode cell setup. Initially, cyclic voltammetry (CV) was performed at scan rates of 4, 6, 8, 10, 20, 30, 40, and 50 mV s^–1^ within a potential window of −0.6 to +1.0 V vs saturated Ag/AgCl. The charge storage kinetics were analyzed by determining the *b*-value, which describes the relationship between the peak current (*i*_p_) and the scan rate (*v*) according to the power law equation (eq 3),^43^ where *a* and *b* are adjustable parameters. By expressing this relationship in logarithmic form, a linear equation is obtained (eq 4). The *b*-value is determined from the slope of the linear regression plot of log­(*i*_p_) vs log­(*v*). A *b*-value of 0.5 indicates a diffusion-controlled process, whereas a value of 1.0 suggests a surface-controlled (capacitive) charge storage mechanism.

3 $$i_{p}=av^{b}$$

4 $$\log\left(i_{p}\right)=\log\left(a\right)+b\;\log\left(v\right)$$

Afterward, galvanostatic charge–discharge (GCD) tests were performed at current densities of 200, 300, 400, 500, 600, and 200 mA g^–1^, acquiring 7 cycles per step, within a potential window of −0.6 to +1.0 V vs saturated Ag/AgCl. The cyclic stability test was carried out for 100 cycles at 500 mA g^–1^ within the same potential window. The specific capacity of the material was calculated using eq 5, where *i* is the current density in A g^–1^ and *t* is the time in seconds. The Coulombic efficiency was obtained from the ratio of the discharge capacity to the charge capacity. Electrochemical impedance spectroscopy was performed in 10 mV_rms_ from 10^5^ to 0.01 Hz at OCP potential. All analyses were performed using a μStat-i 400s potentiostat (DropSens/Metrohm), and data acquisition was carried out using the DropView 8400M software (DropSens/Metrohm).

5 $$C\;\left({\mathrm{mAh g}}^{-1}\right)=\frac{it}{3.6}$$

## 3 Results and Discussion

In order to evaluate the role of the carbonized collagen matrix in the synthesis of NWO particles, Fourier transform infrared (FTIR) spectroscopy analyses were carried out. Both syntheses, one containing only the niobium precursor and the other containing both niobium and tungsten precursors, were evaluated. Initially, the spectra of the carbonized samples (Figure 2(a),(b)) were obtained. In general, the difference between the spectra was limited to the peaks related to the fingerprint region (<1,000 cm^–1^), doubling the number of peaks observed in Figure 2(b) compared to that in Figure 2(a) (912 to 748 cm^–1^), suggesting the presence of a distinct oxidized metal compound.

**Figure 2:**
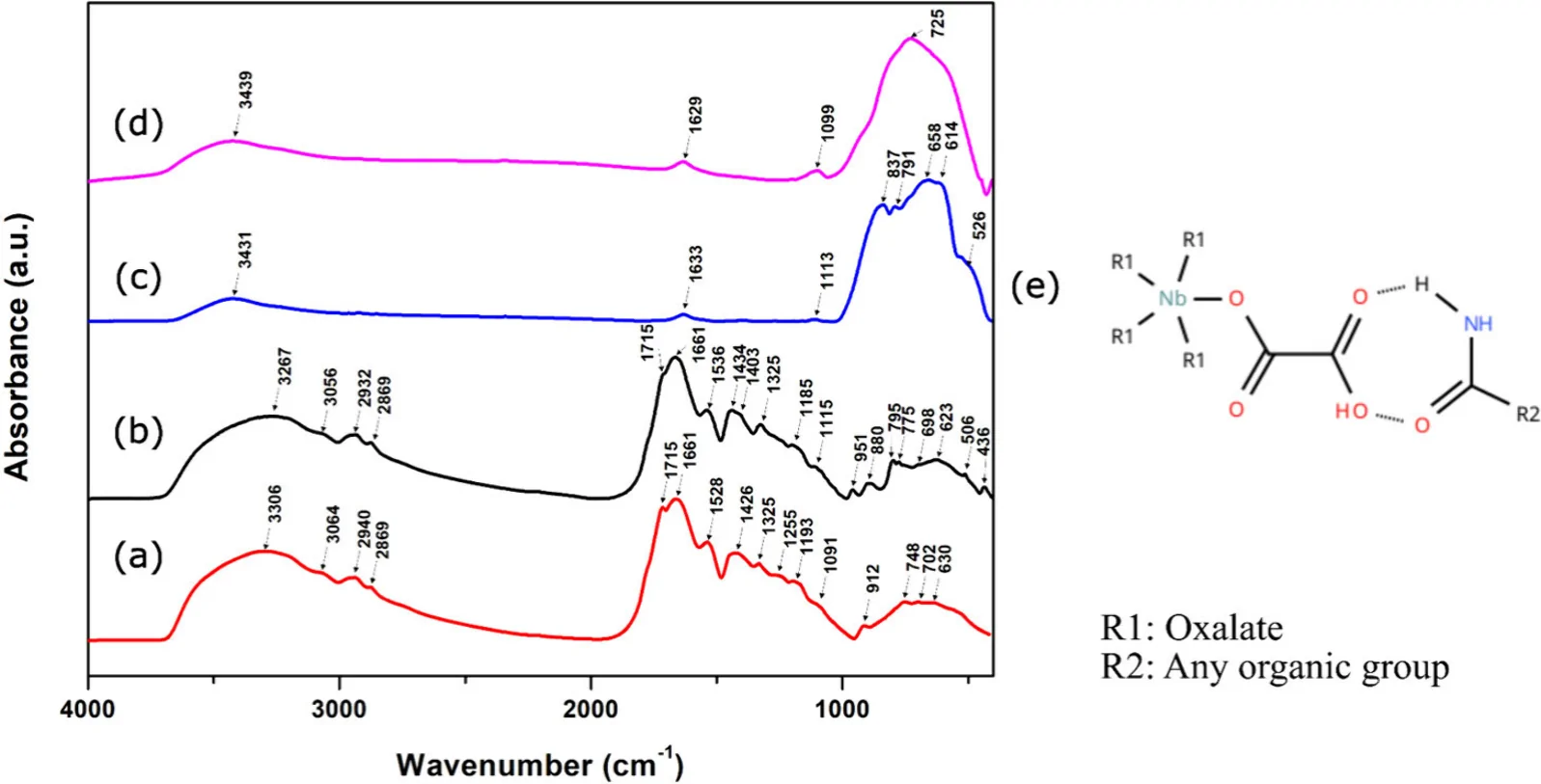
FTIR absorption spectra of (a) carbonized Nb-oxide control sample (NbO), (b) charred mixed Nb–W oxide (NWO), (c) calcined NbO, and (d) calcined NWO. (e) Schematic representation of the dimeric structure formed by the interaction between ammonium niobium oxalate (OANb) and amide groups.

Another relevant observation in both spectra is the presence of protein functional groups (>1,000 cm^–1^), even after the carbonization process. In the analysis of the carbonized samples, the broad band observed at 3,298 cm^–1^ is related to N–H stretching vibrations. In the study by Muyonga et al.,^44^ different FTIR spectra of gelatins obtained from bones and scales of young and adult fish, as well as from different extraction temperatures, were analyzed. In that work, the N–H bonds were associated with a peak centered around 3,400 cm^–1^. However, according to Kemp,^45^ the coupling of the N–H bonds with C=O bonds shifts the absorption band to lower wavenumbers.

Considering that the niobium precursor is an oxalate compound, the observation of such behavior suggests a strong interaction between the protein chains and the metal complex within the carbonaceous matrix. Another relevant point is the overlapping of the N–H stretching band with peaks associated with the CH_2_ group (3081, 2943, and 2873 cm^–1^). According to the same author,^45^ this behavior indicates the formation of a dimeric intermolecular bond between amide groups and a carboxylic acid (Figure 2(e)), reinforcing the formation of a network between the niobium complex and the collagen chains.

Another peak, with negligible intensity and located at 2,220 cm^–1^, is related to the formation of nitrile groups (C≡N). When present in significant amounts, this group exhibits an intense band in the 2,300 to 2,200 cm^–1^ region;^45^ however, given the low intensity observed here, only trace amounts are expected. Two peaks associated with different carbonyl groups were observed at 1,724 and 1,668 cm^–1^, the former being attributed to the oxalate carbonyl and the latter to the amide group.^45,46^

The peak at 1,534 cm^–1^ is attributed to N–H stretching coupled with C–N bending vibrations. Additionally, CH_2_ bending vibrations were observed at 1,425 cm^–1^, while the peak at 1,331 cm^–1^ corresponds to the CH_2_ wagging vibrations of proline, within the amide II region.^44^ At 1,265 cm^–1^, a shoulder is observed, which is commonly associated with the C–O vibrations of carboxylic acid C–OH bonds.^47^

Following calcination, the spectra of the pure oxides were also analyzed. In Figure 2(c),(d), for both systems (Nb and Nb/W), there is a general suppression of organic functional groups peaks. In Figure 2(c), the spectrum of the niobium-only compound exhibits characteristic peaks of the metal oxide and peaks related to the presence of structural water in the material (3,423 and 1,629 cm^–1^).^48^ At 849 cm^–1^, a peak corresponding to the Nb–O–Nb stretching mode is observed,^49^ while Nb_3_O vibrations appear at 537 cm^–1^;^49^ Nb–O–Nb bridging modes are detected in the 750–580 cm^–1^ range.^49^

However, in the spectrum of the mixed oxide (Figure 2(d)), a marked change in the vibrational profile is evidenced when compared with the niobium-only sample. Most of the vibrational features collapse into a single dominant band at 725 cm^–1^. In the work of Delgado et al.,^50^ ammonium metatungstate and niobium oxalate were used as precursors in hydrothermal synthesis with varying Nb/W atomic ratios. A notable observation in that study was the progressive modification of the niobium oxide FTIR spectrum with increasing tungsten content, with most peaks of pure niobium oxide converging into a single band at approximately 800 cm^–1^.

Therefore, the merging of the vibrational signals observed for the NWO sample suggests the incorporation of tungsten into the oxide compounds. In light of these findings, XRD and Rietveld refinement analyses of NWO were conducted to determine whether a homogeneous mixed structure or a heterojunction of tungsten and niobium oxides was formed.

The X-ray powder diffractogram of NWO is shown in Figure 3(a). From a preliminary analysis, the diffraction peaks were located at positions similar to those reported for compounds such as NaNbO_3_ (COD-1011064) and NaWO_3_ (COD-1010419).^51–59^ Based on these comparisons, Rietveld refinement was performed in order to determine the Nb:W atomic ratio within the crystal structure. Reference structures were used: ICSD 028588-cubic^60^ for the main phase and ICSD 164626 ^61^ as the minority isostructural analogue. The fit between the experimental and calculated diffractograms is presented in Figure 3(b).

**Figure 3:**
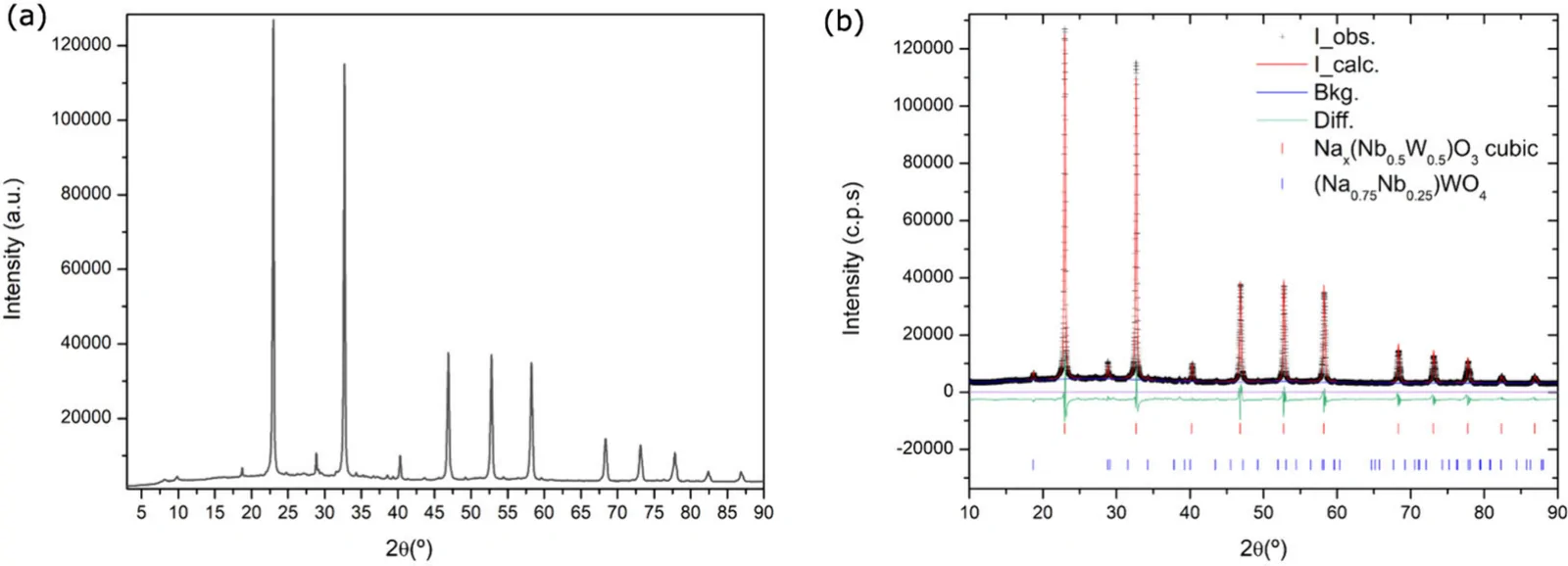
(a) XRD pattern of the mixed Nb–W oxide (NWO) sample measured at 0.5° min^–1^ and (b) corresponding Rietveld refinement results of Na_*x*_(Nb_0.5_W_0.5_)­O_3_ and (Na_0.75_Nb_0.25_)­WO_4_.

As a result, a major phase corresponding to Na_*x*_(Nb_0.5_W_0.5_)­O_3_ was identified, accounting for 96.74% of the sample. This phase crystallizes in a cubic structure with space group *Pm*3̅*m*, a lattice parameter of *a* = 3.88211(6) Å, and an average crystallite size of 162(2) nm. A minor phase (3.26%) was also detected and assigned to (Na_0.75_Nb_0.25_)­WO_4_, which crystallizes in a tetragonal structure with space group *I*4_1_/*a*, lattice parameters *a* = *b* = 5.238(2) Å and *c* = 11.359(3) Å, and a crystallite size of 200(30) nm. For the main phase, the Nb:W ratio was fixed at its nominal 50:50 value, since it is strongly correlated with the phase scale factor and cannot be determined independently from these data. Statistical parameters such as the weighted profile *R*-factor (*R*_wp_ (%) = 7.42), goodness of fit (GOF = 5.39), number of observations (*N*_obs_ = 8001), and number of variables (*N*_var._ = 31) together with the graphical profile of Rietveld refinement demonstrated a great fit between the experimental and simulated structure. The *R*(Bragg) value of the impurity phase was used solely as a qualitative indicator due to limitations in counting statistics. The minority phase A-site occupancy was fixed for structural charge neutrality and not refined. These results demonstrate that the synthesis process using a collagen matrix is effective for producing mixed-metal ceramics. The structural parameters obtained from the refinement are summarized in SI Tables S1–S3.

In a previous study, with the same synthesis method as that used for NWO, Nb_2_O_5_ nanoparticles were obtained.^34^ However, the crystallite size values suggested an average size very close to or even exceeding the instrumental resolution limit of the Scherrer equation. Assuming an initially isotropic crystalline shape, this result would indicate the absence of true nanostructures, with the material instead being quasi-nanostructured. Another relevant aspect is that the structural similarity to stoichiometric perovskites, such as NaWO_3_, implies the presence of tungsten in the +5 oxidation state. This would limit the total electrochemical capacity of a potential electrode material since W^6+^ could accommodate a larger number of electrons. Owing to the uncertainty regarding the particle shape and size, FEG-SEM analysis was therefore performed.

Figure 4 and the Supporting Information (Figure S2) reveal the morphology of the obtained particles. Most particles exhibit a cubic shape with a small fraction presenting ellipsoidal morphologies. In agreement with the predominant crystalline symmetry, natural nucleation is expected to favor the formation of cubic particles. However, this observation highlights an important aspect of the particle formation process. Although a rigid matrix is generated during the carbonization step of the synthesis, it is insufficient to exert strict control over the particle shape, which instead follows the crystalline arrangement of the material.

**Figure 4:**
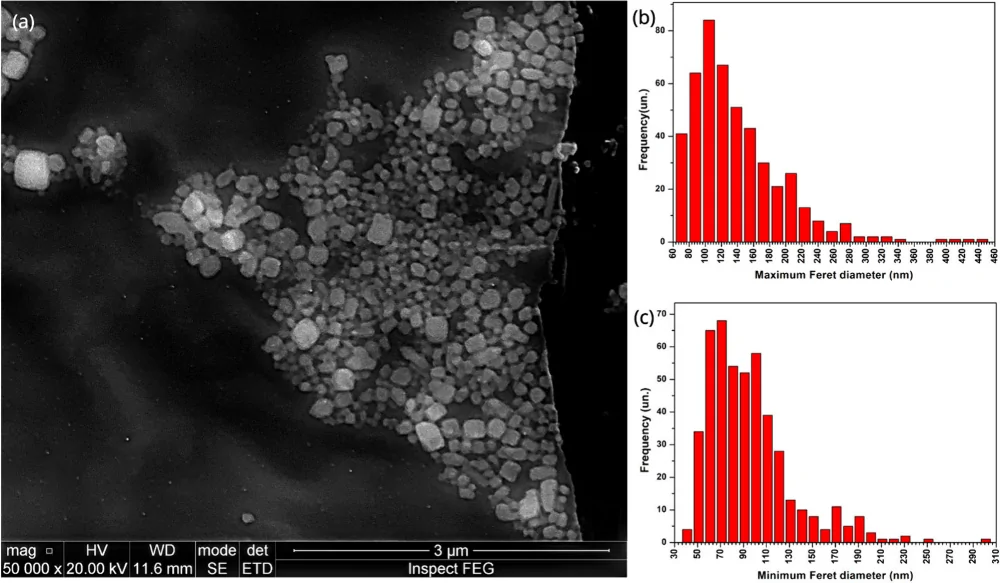
(a) FEG-SEM micrograph of the mixed Nb–W oxide (NWO) obtained at 20 keV and 50k× in addition to histograms of the (b) largest and (c) smallest Feret diameters of the particles.

From the acquired images, the dispersion of particle dimensions was evaluated using Feret diameters.^38^ Accordingly, the largest and smallest Feret diameters were measured. The histograms corresponding to these distributions are shown in Figure 4, based on the analysis of 470 particles.

The largest Feret diameters (Figure 4(b)) include values ranging from approximately 62 to 441 nm, with a mean of approximately 138 nm and a median of approximately 124 nm, indicating a positively skewed distribution. This suggests that, while most particles are concentrated at lower diameter values, a limited number of larger particles shift the mean toward higher values.

Figure 4(c) presents the distribution of the smallest Feret diameters of NWO. The average value was approximately 95 nm, with a median of approximately 88 nm, again indicating a right-skewed distribution. The minimum and maximum values observed were 36 and 304 nm, respectively.

The coefficients of variation were approximately 41% for the largest Feret diameters and 39% for the smallest Feret diameters, indicating that the collagen-based dispersion synthesis method yields a highly polydisperse population of particles. Particle size reduction is a conventional approach to shorten diffusion pathways and enhance the ion intercalation kinetics. Although some particles larger than 200 nm are present, their fraction is low; thus, a significant amount of inactive mass in the final electrode is not expected.

Additionally, the form factor calculated from the average particle diameters was 1.46, suggesting a slightly elongated morphology and deviation from an isotropic shape. Finally, as more than 50% of the particles exhibit a smallest Feret diameter below 100 nm, the material can be classified as a nanostructure.^62^ A summary of the statistical parameters is presented in SI Table S4.

For the application of the material as an electrode in sodium-ion batteries, it was important to maintain tungsten in the +6 oxidation state. When comparing the capacities of the material, the contribution related solely to the sodium extraction/intercalation process would be limited, yielding a value of approximately 68 mAh g^–1^. However, when including the redox contribution associated with the reduction of Nb^5+^ and W^5+^, a theoretical maximum capacity of 203.2 mAh g^–1^ (SI eq S1) is expected, assuming reduction to common oxidation states such as Nb^4+^ and W^4+^. With an additional reduction step involving tungsten, a capacity of 270.9 mAh g^–1^ (SI eq S2) could be achieved, representing an increase of 33.3% in the total capacity. In order to evaluate this point, the X-ray photoelectron spectroscopy (XPS) analysis of the material was conducted. Figure 5(a) shows the survey XPS spectrum of the NWO powder sample, in which Nb, W, O, C, and Na are identified, as expected, with no detectable impurities. The Nb 3d XPS region exhibits a single Nb^5+^ component at 207.5 eV, consistent with the presence of Nb_2_O_5_. The W 4f XPS region shows a dominant component at 36.1 eV, attributed to W^6+^, along with a minor contribution from W^4+^, consistent with WO_3_ and WO_2_, respectively.

**Figure 5:**
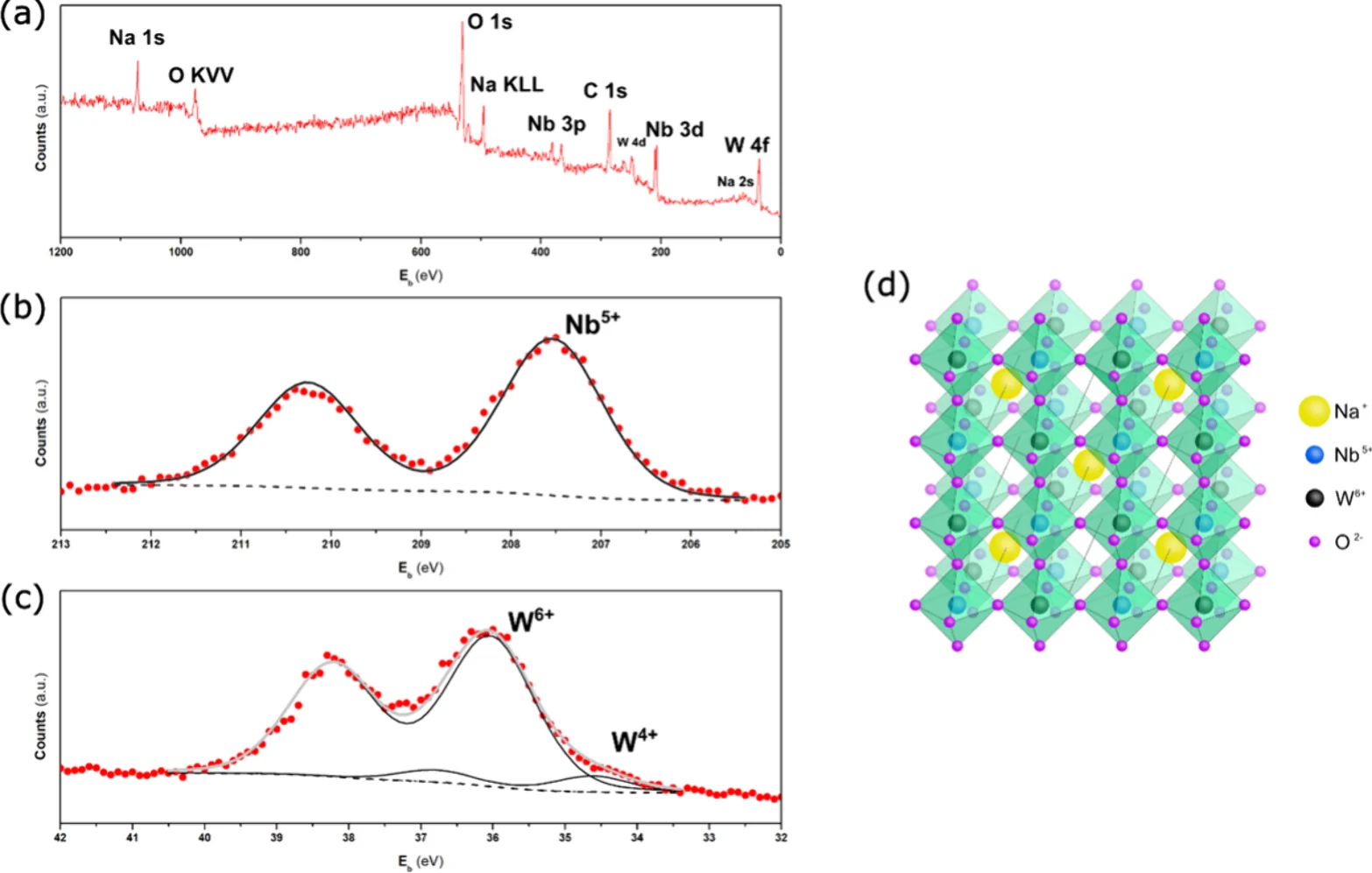
(a) Mixed Nb–W oxide (NWO) survey XPS spectrogram, (b) NWO Nb 3d high-resolution XPS spectrum, (c) NWO W 4f high-resolution XPS spectrum, and (d) proposed crystal structure for NWO Na_0.5_(Nb_0.5_W_0.5_)­O_3_. Solid lines in (b) and (c) represent the fitting results using symmetric Gaussian–Lorentzian sum functions after Shirley-type background subtraction.

The W 4f XPS measurements of the NWO sample point to 8% W^4+^ + 92% W^6+^. The presence of W^4+^ cannot be attributed to X-ray exposure because the NWO samples in Figure 7(c) were measured under the same conditions and no W^4+^ signal was achieved. The probed depth in these XPS measurements was 6 nm (λ = 2 nm), so the W^4+^ component is present in this region close to the surface, probably coming from a surface defect. In conclusion, there is no substantial effect on electrochemical charge storage.

Thus, although the crystalline arrangement induces the formation of geometries similar to those of NaWO_3_, in which tungsten is typically in the W^5+^ state, tungsten in the present material is predominantly stabilized as W^6+^. Consequently, to preserve charge neutrality without altering the oxidation state of niobium, the structure must accommodate the sodium vacancies within the crystal lattice. This finding supports the hypothesis that the final material composition aligns with the Na_0.5_(Nb_0.5_W_0.5_)­O_3_ stoichiometry, thereby validating our main objective of successful tungsten incorporation. Based on the integration of diffraction data and the inferred ionic configuration of the lattice, it can be concluded that the material exhibits a crystalline arrangement consistent with the structural model illustrated in Figure 5(d).

During the electrode activation process, an increasing trend in voltammetry area was observed in 1 M NaOH at a scan rate of 50 mV s^–1^ (Figure 6(a)). Since the voltammetric area is proportional to the total electrical charge involved in an electrochemical process, a substantial increase in charge storage capacity was observed over successive cycles (SI Figure S3), in both the anodic (Figure 6(b)) and cathodic (Figure 6(c)) directions. When comparing the electrode performance in 1 M Na_2_SO_4_ + 0.8 mM SDS before and after activation, a pronounced change in the voltammetry profiles is evident (Figure 6(d)), accompanied by significantly higher current densities. Consequently, this treatment was defined as an activation process and was adopted as the standard procedure for all subsequent electrochemical measurements. Furthermore, to elucidate the mechanisms underlying material activation, XPS analyses of the electrode were performed before and after the activation process.

**Figure 6:**
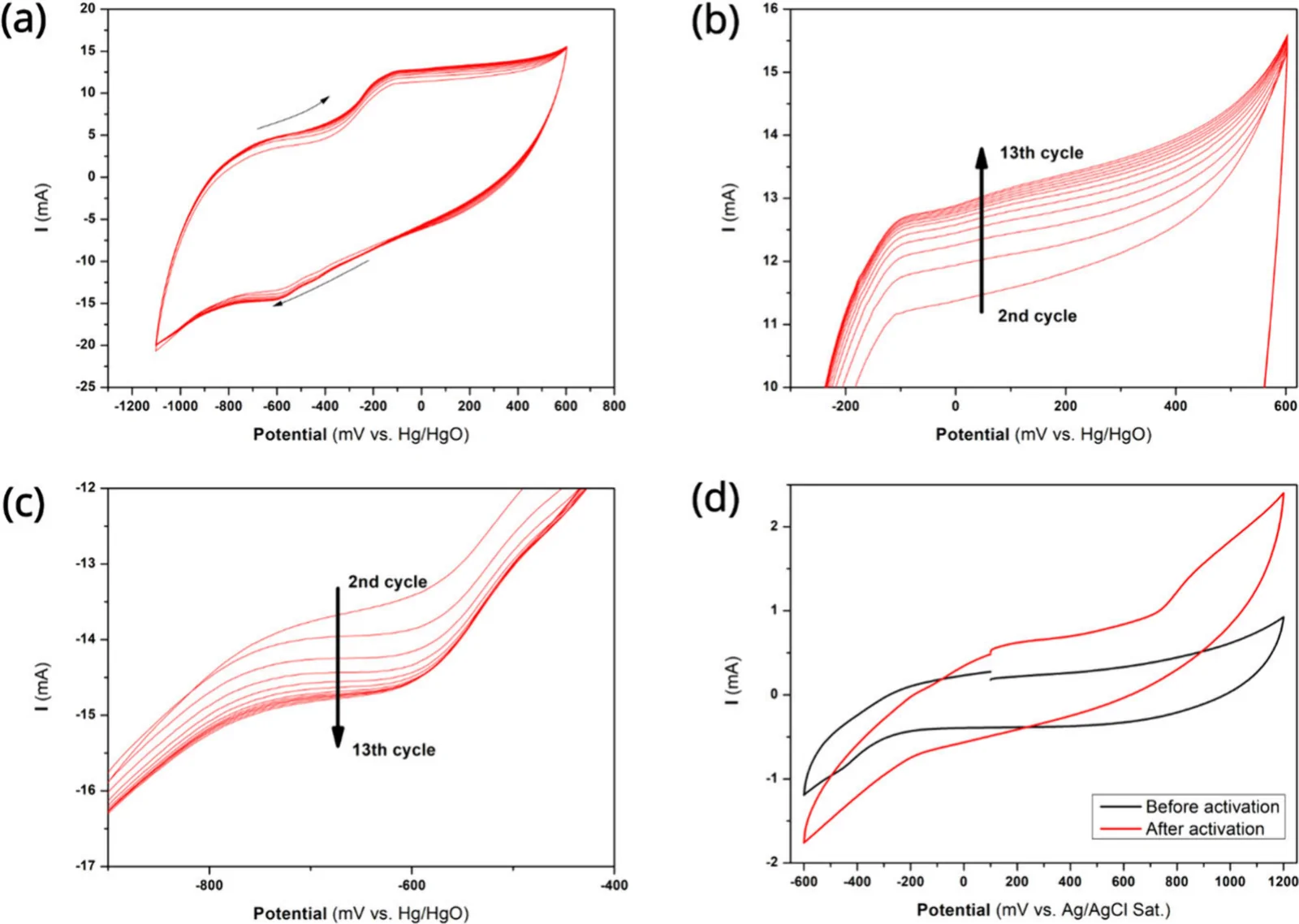
Electrochemical activation process of mixed Nb–W oxide (NWO) in 1 M NaOH at 50 mV s^–1^ over 13 cycles: (a) full cyclic voltammograms; (b) magnification of the anodic region; (c) magnification of the cathodic region; and (d) comparative cyclic voltammograms at 50 mV s^–1^ in 1 M Na_2_SO_4_ + 0.8 mM SDS before (black) and after (red) the activation process.

Figure 7(a) shows the survey XPS spectra of the NWO electrode before and after activation. In contrast to the raw NWO powder, fluorine is detected in the electrodes, which is attributed to the binder used in the electrodes (PVDF) and the conductive additive (Vulcan). The Nb 3d region (Figure 7(b)) does not show significant changes compared to the NWO powder, presenting a single Nb^5+^ component, as expected.

**Figure 7:**
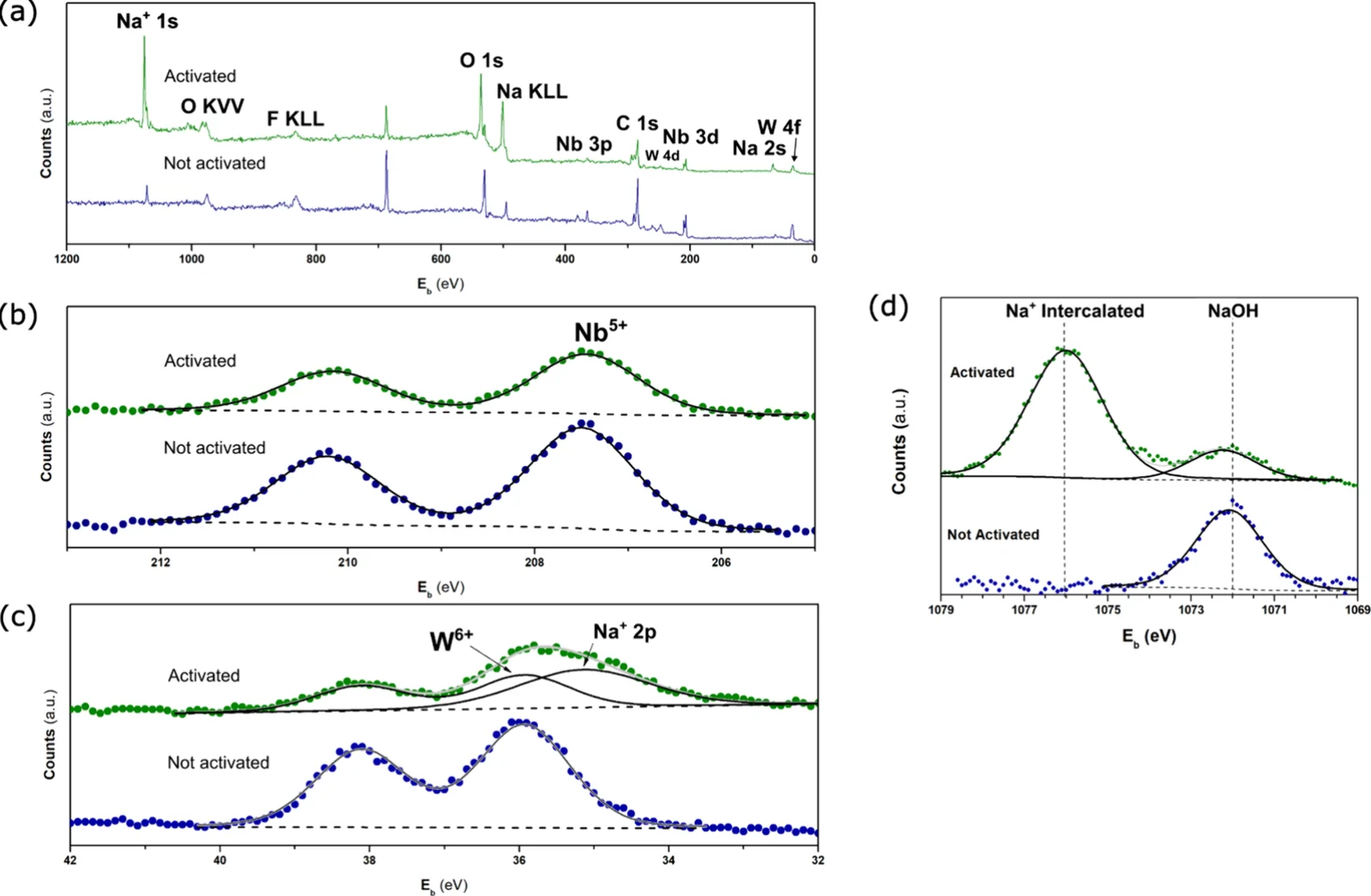
XPS spectra at the (a) survey, (b) Nb 3d, (c) W 4f, and (d) Na 1s regions of the mixed Nb–W oxide (NWO) electrodes samples. Solid lines represent the fitting results using symmetric Gaussian–Lorentzian sum functions after Shirley-type background subtraction.

In contrast, the W 4f XPS region (Figure 7(c)) exhibits significant changes after electrode activation. While the pristine electrode shows only the W^6+^ component, an additional contribution appears after activation. This feature does not belong to the characteristic W 4f doublet but is instead attributed to overlap with the Na 2p region. The Na 2p signal, typically located at lower binding energies (∼31 eV),^1,63^ indicates the presence of sodium in a distinct and highly polarized chemical environment.

This interpretation is corroborated by the Na 1s XPS analysis (Figure 7(d)). Before activation, the Na 1s spectrum shows a single component at 1072.0 eV, attributed to Na–O bonding.^64^ After activation, a new component emerges at 1076.1 eV, which is assigned to sodium incorporated into the (Nb_0.5_W_0.5_)­O_3_ lattice.

Taken together, these results provide strong evidence that the electrode activation process is mediated by sodium insertion into structural vacancies of the material and that the charge storage mechanism occurs predominantly through intercalation. To investigate the underlying charge storage mechanism in the material, we conducted cyclic voltammetry (CV), galvanostatic charge–discharge (GCD), and electrochemical impedance spectroscopy (EIS) measurements.

The cyclic voltammetry profile evidences the dominant charge storage mechanism of the material.^43^ To evaluate the electrochemical behavior over a wider potential window, an electrolyte containing 1 M Na_2_SO_4_ was selected, as in NaOH, the system reached the oxygen evolution potential at values above 0.6 V vs Hg/HgO. As shown in Figure 8(a),(b), four distinct peaks were observed at 0.029 (I), 0.27 (II), −0.127 (III), and 0.21 (IV) V vs saturated Ag/AgCl at 10 mV s^–1^, where the measured *b*-values were 0.774, 0.664, 0.581, and 0.667, respectively, all exhibiting *R* ^2^ > 0.99. Since *b*-values near 1 indicate a purely capacitive mechanism and values near 0.5 indicate diffusion-controlled kinetics, these intermediate values point to a hybrid system with a predominant diffusion-controlled component (*b* < 0.75), typical of intercalation pseudocapacitance. Furthermore, the cyclic voltammetry profile, which lacks both the classic rectangular shape and highly defined redox peaks (Figure 8(a)), supports the hypothesis of a mixed-controlled charge storage mechanism. In addition to cyclic voltammetry, galvanostatic charge–discharge (GCD) measurements were performed at 200, 300, 400, 500, 600, and 200 mA g^–1^ (Figure 8(e)), yielding specific capacities of 243, 158, 115, 83, 58, and 242 mAh g^–1^, respectively. Regarding cycle retention, the electrode maintained 99.7% of its capacity after 100 cycles at 500 mA g^–1^ (Figure 8(f)), demonstrating high initial stability. Compared to other state-of-the-art cathode materials for aqueous sodium-ion batteries (ASIBs), NWO exhibited superior performance in terms of specific capacity at moderate current densities (0.2–0.4 A g^–1^), highlighting its promise for energy storage applications (Table 1). Upon analyzing the charge–discharge profiles, it is evident that the process lacks the characteristic triangular shape expected in electric double-layer capacitors, yet it does not display the flat plateaus typical of purely faradaic systems, thus demonstrating classic hybrid pseudocapacitive behavior.

**Figure 8:**
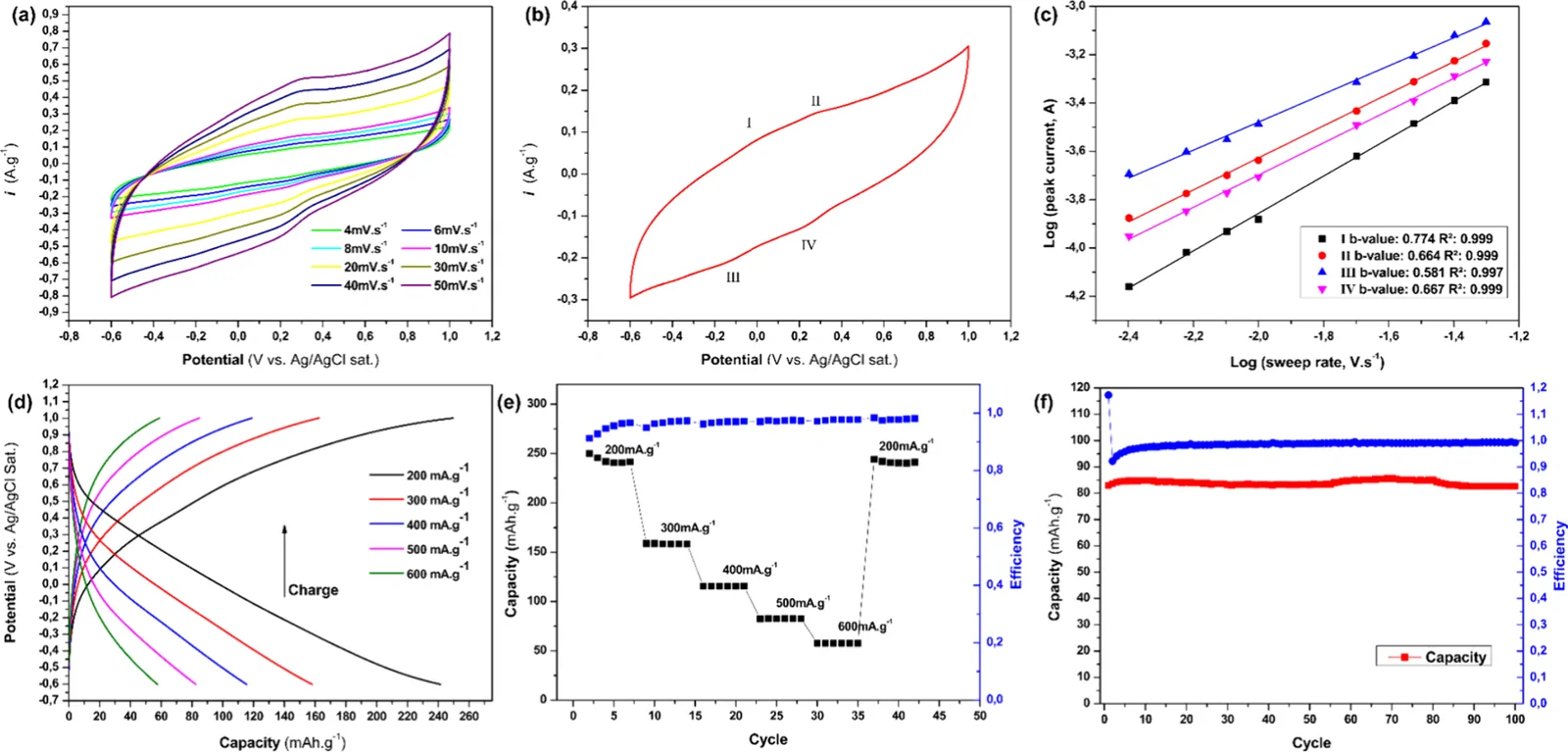
Electrochemical measurements in 1 M Na_2_SO_4_: (a) cyclic voltammetry curves at 4, 6, 8, 10, 20, 30, 40, and 50 mV s^–1^; (b) representation of the peak positions observed in the cyclic voltammogram at 10 mV s^–1^; (c) *b*-value determination via linear regression of the log of peak current vs the log of scan rate; (d) charge–discharge profiles of the NWO electrode; (e) rate capability performance at current densities of 200, 300, 400, 500, and 600 mA g^–1^; and (f) cyclic stability test at 500 mA g^–1^ over 100 cycles.

**Table 1:** Comparison of the Electrochemical Performance of the NWO Electrode with Other State-of-the-Art Cathode Materials for Aqueous Sodium-Ion Batteries

Material	Electrolyte	Capacity (mAh g^–1^)	*i* (A g^–1^)	Cycles/Ret (%)	Year	Ref.
Na_0.5_(Nb_0.5_W_0.5_)O_3_	1 M Na_2_SO_4_	243, 158, 115, 83, and 58	0.2, 0.3, 0.4, 0.5, and 0.6	100/99.7%	2026	This Work
Na_3_VMn_0.5_Ti_0.5_(PO_4_)_3_	2 M NaAc + 1 M ZnAC	59.6, 55.9, 51.1, 48.7, 45.8, 38.1, 23.4, 17.4, and 11.5	0.02, 0.04, 0.1, 0.14, 0.2, 0.4, 1, 1.4, and 2	400/70.6%	2025	^65^
NaMnO_2_	1 M Na_2_SO_4_	102, 94, 90, 90, 83, and 80	1, 2, 3, 4, and 5	100/96%	2025	^66^
Na_3_V_1.3_Cr_0.7_(PO_4_)_3_@C (NV_1.3_Cr_0.7_P)	1 M Na_2_SO_4_	61, 59, 58, 56, 55, 52, and 48	0.1, 0.2, 0.5, 1, 2, 5, and 10	5000/74.8%	2025	^67^
Na_0.54_MnO_2_	Sat. NaNO_3_	72.6, 66.8, and 57.5	1, 2, and 5	-	2025	^68^
MnPBA@NiHCF	1 M NaCl	70.8, 65.5, 60.9, 54.9, 51.1, 49.6, 47.6, and 45.2	0.5, 1, 2, 5, 8, 10, 12, and 15	20000/95.3%	2025	^69^
Na_1.88_Mn[Fe(CN)_6_]_0.97_	17.0 M NaClO_4_ + 0.33 M Na_4_Fe(CN)_6_	180, 112, 72, and 49	0.1, 0.2, 0.5, and 1	800/62%	2025	^70^
Na_0.71_MnO_2_·0.15H_2_O	1 M Na_2_SO_4_	176	0.2	60000/90.5%	2025	^71^
Ni@FeHCF-X	1 M Na_2_SO_4_	75, 56, 42, 36, and 32	0.1, 0.2, 0.3, 0.4, and 0.5	200/42.16%	2024	^72^
Na_0.16_Ba_0.08_Mn_0.92_O_2_·0.5H_2_O	17.0 M NaClO_4_	117.2, 99.9, 92.4, 85.5, 77.8, 65.3, and 53.1	0.1, 0.2, 0.5, 1, 2, 5, and 10	10000/102%	2024	^73^
Na_1.36_Ni_0.4_Fe_0.15_Co_0.15_Mn_0.15_Cu_0.15_[Fe(CN)_6_]	17.0 M NaClO_4_	118.6, 109.8, 103.2, 87.4, 72.6, 57.7, and 46.3	0.1, 0.2, 0.5, 1, 2, and 5	1800/81.2%	2024	^74^

Another tool used to achieve a comprehensive understanding of the system was electrochemical impedance spectroscopy (EIS). From the Nyquist plot (Figure 9(a)), it was possible to identify the equivalent series resistance (*R*_s_) of the system as 6.046 Ω and the charge transfer resistance (*R*_ct_) as 0.48 Ω. While the former component falls within typical values for Na_2_SO_4_ electrolytes, the latter indicates that the charge transfer process occurs without significant hindrance. This is a direct indication that the insertion of a different-valence ion (W^6+^) into the sodium niobate structure (Nb^5+^) leads to high surface charge transfer kinetics.

**Figure 9:**
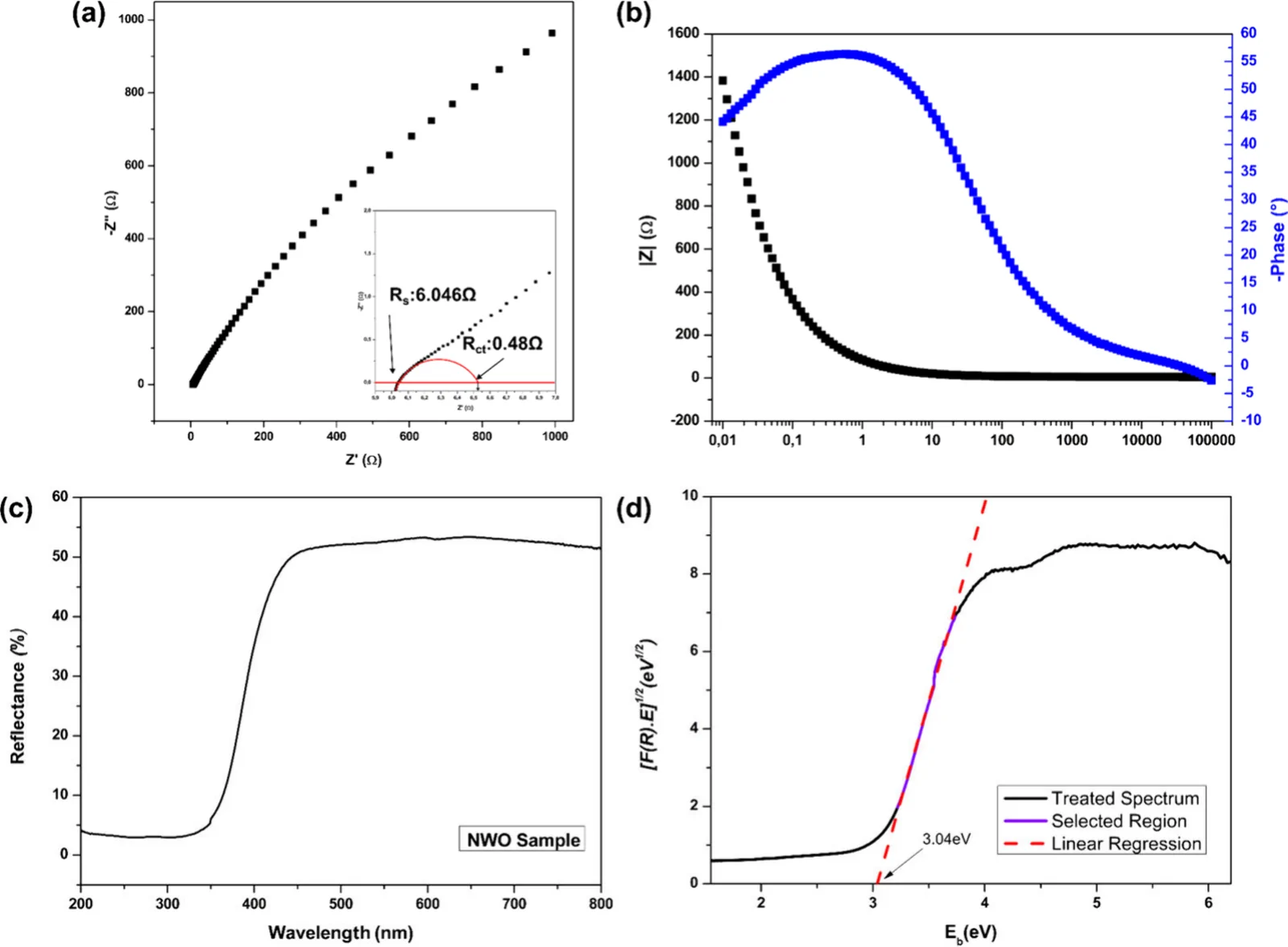
(a) Nyquist and (b) Bode plots of NWO electrode in 1 M Na_2_SO_4_. (c) reflectance spectrum and (d) Tauc plots of NWO powder obtained by DRS.

Furthermore, the Bode plot (Figure 9(b)) corroborated the behavior observed in the previous electrochemical tests. Analyzing the negative phase angle profile, the dominant energy storage process exhibits stability at around 55° at low frequencies (10 to 0.1 Hz) with an impedance modulus below 300 Ω. In other words, the electrode exhibits both capacitive and diffusion-controlled processes but with a greater prominence of the latter. From this perspective, the material was analyzed by UV–vis diffuse reflectance spectroscopy (DRS), since as a semiconductor its conductivity is closely related to its bandgap energy (*E*_b_).^75^ This property corresponds to the energy difference between the top of the valence band and the bottom of the conduction band. Consequently, a reduction in this energy gap implies that lower energies are required for electronic conduction.

Based on the treatment of the experimental data, the optical bandgap was determined using the Tauc plot method (Figure 9(d)) of the reflectance spectrum (Figure 9(c)). Linear regression of the appropriate region yielded an *E*_b_ value of 3.04 eV. When compared to the commonly reported value of 3.4 eV^1^ for NaNbO_3_, this represents a reduction of approximately 11%. This decrease suggests enhanced charge transfer kinetics in the material due to tungsten incorporation. Previous studies have demonstrated that increasing the WO_3_ proportion in a Nb_2_O_5_ thin film from 10% to 20% reduced the bandgap energy by 11.46%.^76^ Furthermore, the calcination temperature has also been reported to influence this parameter.^77^

## 4 Conclusions

On the basis of the results presented in this study, it can be concluded that the collagen dispersion method enables the successful fabrication of a mixed Nb–W perovskite. Moreover, the use of a tungsten anionic complex (WO_4_^2–^) allowed the synthesis to be carried out in an aqueous medium without precipitate formation. FTIR analysis demonstrated that the nanostructure formation through this route is mediated by strong interactions between the ammonium niobium oxalate (ANO) precursor and the amino acid chains of the protein, indicating that the process is not purely physical in nature.

Regarding the general characteristics of the obtained material, the FTIR spectrum of NWO exhibited distinct vibrational features when compared to the NbO, with peak coalescence indicating the prominence of a specific bonding environment. XRD characterization further demonstrated that the synthesized material represents an intermediate structure between NaNbO_3_ and NaWO_3_ perovskites, confirming a Na_*x*_(Nb_0.5_W_0.5_)­O_3_ stoichiometry in 97% of the sample, with a minor contribution (3%) from the (Na_0.75_Nb_0.25_)­WO_4_ phase. The cubic crystalline arrangement strongly influenced particle morphology, indicating that the carbonaceous collagen matrix primarily controls particle size, yielding a mean minimum Feret diameter of approximately 95 nm. XPS analysis revealed that the high proportion of Nb^5+^ stabilizes tungsten predominantly in the W^6+^ oxidation state, leading to a higher theoretical capacity (∼270 mAh g^–1^) and the formation of sodium vacancies within the structure. These vacancies suggest a final stoichiometry of Na_0.5_(Nb_0.5_W_0.5_)­O_3_ to preserve the overall charge neutrality of the crystal lattice.

Finally, XPS analysis revealed that the electrode activation process in 1 M NaOH is likely driven by the intercalation and deintercalation of sodium ions within the material’s lattice. The absence of metallic site reduction, coupled with an increase in the binding energy of structural oxygen, indicates that charge storage is governed by intercalation-type pseudocapacitance. Furthermore, electrochemical evaluations elucidated the underlying interfacial processes, confirming that NWO is a highly promising electrode candidate for aqueous sodium-ion batteries due to its high capacity (243 mAh g^–1^ at 200 mA g^–1^) at a relative high current density. This behavior is largely attributed to a probable increase in the electrical conductivity of the material, associated with a bandgap of 3.04 eV, representing a reduction of approximately 11% compared to that of NaNbO_3_, and a low *R*_ct_ (0.48 Ω).

Overall, the new green synthesis route based on collagen dispersion represents a viable technique for obtaining nanostructures, not only pure oxides but also mixed oxide systems. Furthermore, NWO demonstrated promising potential for electrical energy storage applications, with strong sustainability appeal, from its synthesis to its final application.

## Supplementary Material



## Notes

Detailed methodology for precursor purification, thermal analysis (TGA/DTG) of the charred precursor, structural and Rietveld refinement parameters including atomic coordinates, FEG-SEM micrograph and statistical distribution of particle diameters, theoretical capacity calculations, and cyclic voltammograms detailing the electrochemical activation process (PDF)
