# Supplementary material for: A Novel Mixed Perovskite-like Alkali Niobium Tungsten Oxide (Na x (Nb0.5W0.5)O3) Synthesized by a Sustainable Collagen Dispersion Process
Source: ACS Omega. 2026 Sep 21;11(39):58752–64. doi: 10.1021/acsomega.6c02992 (PMC13648068; doi:10.1021/acsomega.6c02992)

Supporting Information

A novel mixed perovskite-like alkali niobium tungsten oxide (Nax(Nb0.5W0.5)O3) synthesized by a sustainable collagen dispersion process

Gabriel Luiz Rasch [a], Adilar Gonçalves dos Santos Júnior [a], Ricardo Faccio [b], Fabiano Bernardi [c], Andrés Cuña Suárez [d,e], Antonio Marcos Helgueira de Andrade [c], Leonardo Moreira dos Santos [f], Roberto Hübler [f], Wladimir Hernandez Flores [g], Célia de Fraga Malfatti [a]

[a] *Corrosion Research Laboratory (LAPEC), Engineering School, Federal University of Rio Grande do Sul (UFRGS), Porto Alegre, Rio Grande do Sul, Brazil.*

[b] *Área Física & Centro Nanomat, DETEMA, Facultad de Química, Universidad de la República, Av. Grail. Flores 2124, C.P., 11800, Montevideo, Uruguay*

[c] *Institute of Physics, Federal University of Rio Grande do Sul (UFRGS), Porto Alegre, Rio Grande do Sul, Brazil.*

[d] *Area Fisicoquímica, DETEMA, Facultad de Química, Universidad de la República, Montevideo, Uruguay.*

[e] *Laboratorio de Energías Renovables, IPTP, Facultad de Química, Universidad de la República, Pando, Canelones, Uruguay.*

[f] *Materials and Nanoscience Laboratory, Polytechnic School, Pontifical Catholic University of Rio Grande do Sul, Porto Alegre 90619-900, Rio Grande do Sul, Brazil*

[g] *Federal University of Pampa, Bagé Campus, Avenue Maria Anunciação Gomes de Godoy 1650, 96400-100 Bagé, RS, Brazil*

S1. Methodology of ammonium niobium oxalate hydrate purification

Due to the high quantity of insoluble niobium pentoxide (Nb_2O_5, 21% wt%) in ammonium niobium oxalate hydrate (79% $NH_4[NbO(C_2O_4)_2(H_2O)_2]$.nH2O, CBMM), the precursor was purified by dissolution-crystallization method. First, ANO was dissolved in ultrapure type 1 water (Milli-Q®) until its saturation (~325g.L^{-1}) in 40ºC under magnetic stirring. The second step was the centrifugation of the solution in 4000rpm for 20min. The supernatant was transferred to a sealed beaker and stored in a common refrigerator for 48h. The crystalized material was then washed 3 times with 10mL.g^{-1} of absolute ethanol and let dry in a common oven for 24h in 60ºC. The removal of insoluble components was confirmed by dissolution test. Then, to confirm the niobium content of

purified reagent, the precursor was calcinated at 700ºC for 3h and 5ºC.min^{-1}, resulting in a metal molar content of 2.6 mmol of Nb in 1g of purified ANO (Nb_2O_5 as the main product).

S2. Thermal analysis of charred Nb/W mixed oxide

To investigate the thermal evolution of the charred Nb/W precursor, thermogravimetric analysis (TGA) and derivative thermogravimetry (DTG) were performed using an SDT-600 analyzer (TA Instruments, USA). The sample was analyzed in an alumina crucible under an air atmosphere. The thermal program consisted of heating to 500 °C at 10 °C min^{-1}, followed by an isothermal step for 40 min, a second ramp to 800 °C at 5 °C min^{-1}, and a final ramp to 1000 °C at 10 °C min^{-1}. As shown in Figure S1, substantial mass loss occurred up to 600 °C, followed by mass stabilization at 700 °C.

Figure S1. TGA/DTG curves of the charred Nb/W mixed oxide precursor, obtained at a heating to 500 °C at 10 °C min^{-1}, followed by an isothermal step for 40 min, a second ramp to 800 °C at 5 °C min^{-1}, and a final ramp to 1000 °C at 10 °C min^{-1} in an air atmosphere.

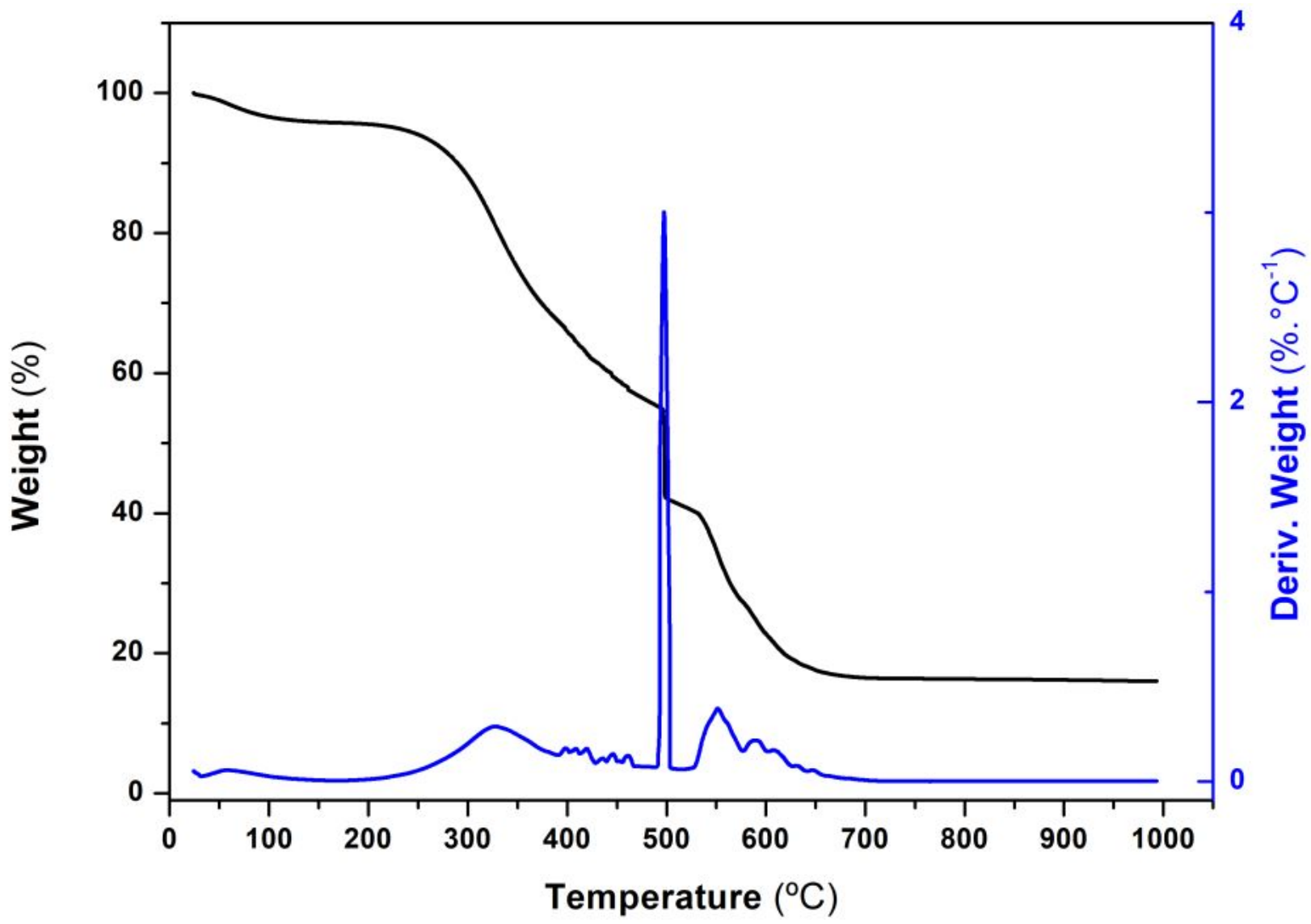

Table S1. Structural and refinement parameters obtained from Rietveld analysis using GSAS-II for a two-phase mixture of $Na_x(Nb_{0.5}W_{0.5})O_3$ and $(Na_{0.75}Nb_{0.25})WO_4$.

	**NaNbWO3 (ICSD 028588-cubic)**	**NaNbWO4 (ICSD 164626)**
Crystallographic data		
Empirical formula	NaNb0.50O3W0.50	Na3NbO16W4
Crystal system	Cubic	Tetragonal
Space group (No.)	P m -3 m (221)	I 41/a (88)
a (Å)	3.88211(6)	5.238(2)
b (Å)	= a	= a
c (Å)	= a	11.359(3)
V (Å³)	58.506(3)	311.7(2)
Z	1	4
Weight fraction (%)	96.74(9)	3.26(9)
ρ_calc (g cm⁻³)	5.616	6.144
Crystallite size (nm)	162(2)	200(30)
Data collection		
Radiation, λ (Å)	X-ray, λ = 1.54051/1.54433	
T (K)	300	
2θ range (min, max, step) (°)	10, 90, 0.01	
Geometry	Bragg-Brentano	
Refinement		
No. of data points	8001	
No. of parameters	31	
background / profile	16 / 3	
No. of restraints	0	
R_p (%)	4.93	
R_wp (%)	7.42	
R_exp (%)	1.38	
R_Bragg (%)	1.47	10.79
S (= R_wp/R_exp)	5.39	
χ²_red	29.05	

Table S2. Atomic coordinates and displacement parameters — NaNbWO3_028588-cubic (NaNbWO3).

**Atom**	**Site**	**x**	**y**	**z**	**U_iso/U_eq (Å²)**	**Occ.**
**Na1**	1 (m3m)	0	0	0	0.039(1)	1
**Nb1**	1 (m3m)	0.5	0.5	0.5	0.0288(5)	0.5
**O1**	3 (4/mmm(z))	0.5	0.5	0	0.026(1)	1
**W**	1 (m3m)	0.5	0.5	0.5	0.0288154	0.5

Table S3. Atomic coordinates and displacement parameters — NaNbWO4_164626-rf (NaNbWO3)

Atom	Site	x	y	z	U_iso/U_eq (Å²)	Occ.
**Na1**	4 (-4(z))	0.5	0.75	0.125	0.03	0.75
**Nb**	4 (-4(z))	0.5	0.75	0.125	0.03	0.25
**W1**	4 (-4(z))	0	0.25	0.125	0.03	1
**O1**	16 (1)	0.2429	0.5969	-0.0383	0.03	1

Table S4. Statistical parameters of the distribution of the largest and smallest diameters of Feret of the mixed Nb-W oxide (NWO) by FEG-SEM.

**Parameter**	**Smallest diameter of Feret**	**Largest diameter of Feret**
Sample quantity (un.)	470	
Average(nm)	95.03	138.97
Median(nm)	87.56	124.49
Maximum(nm)	304.01	441.14
Min.(nm)	36.32	62.65
Standard deviation(nm)	37.39	57.97
Averages Form Factor	1.462	

Figure S2 – FEG-SEM micrograph of NWO deposited on a fluorine-doped tin oxide (FTO) substrate

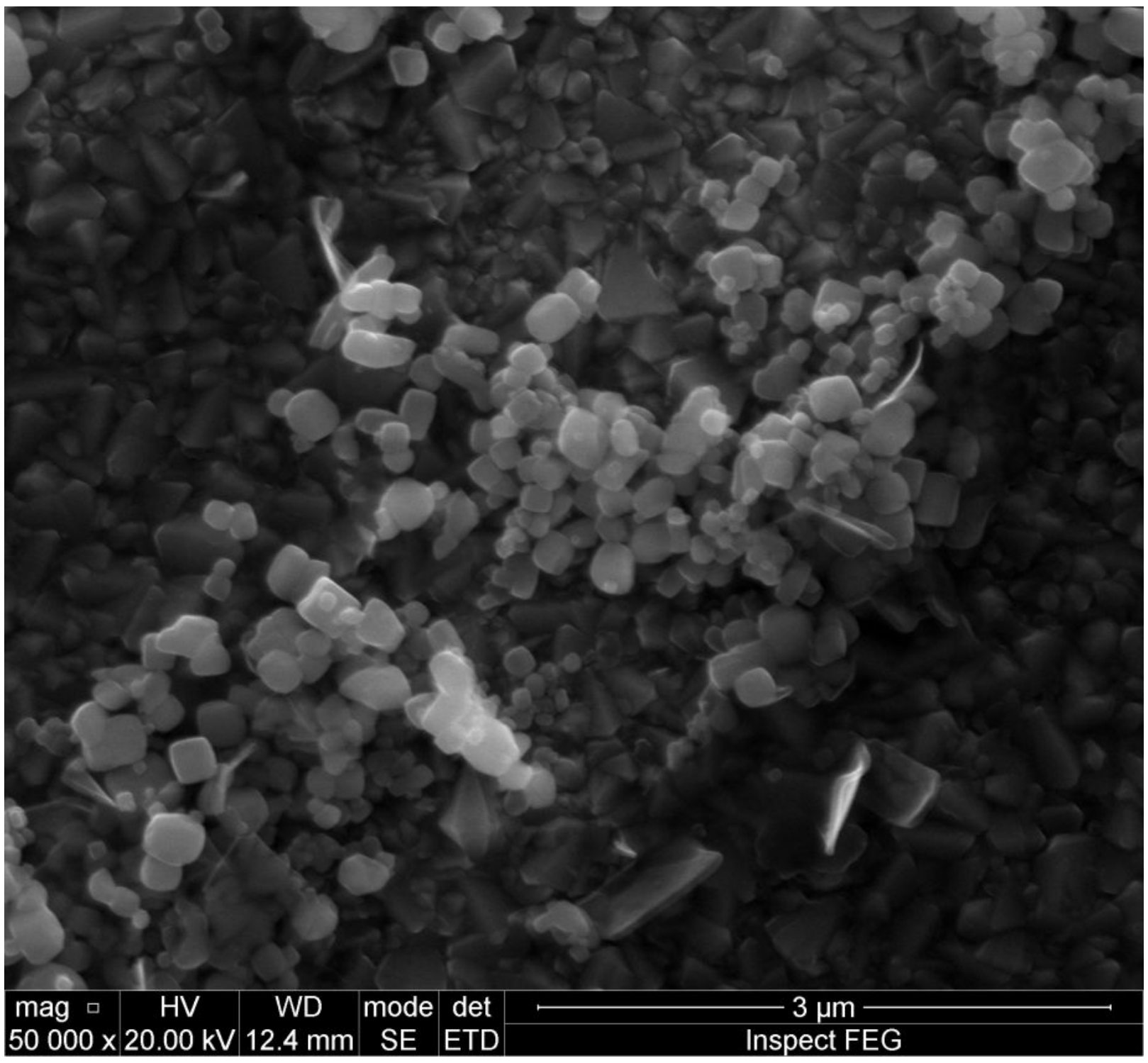

Equation S1. Theoretical capacity of NWO with W^{5+} valence

The theoretical capacity of a material (in $mAh.g^{-1}$) is obtained by multiplying the number of electrons involved in the electrochemical process (n_e) by the Faraday constant (F), and dividing the result by the product of the material's molar mass ($\overline{M}$) and the conversion factor 3.6.

$$\mathrm{C(mAh.}g^{-1}) = \frac{n_e F}{3.6.\overline{M}}$$

$$C = \frac{\left(0.5e_{Na^+} + 0.5e_{W^{5+}\rightarrow 4^+} + 0.5e_{Nb^{5+}\rightarrow 4^+}\right).96{,}485}{3.6\,.\,(0.5 * 23 + 0.5 * 92.9 + 0.5 * 183.84 + 3 * 16)}$$

$$C = 203.2\ mAh.g^{-1}$$

Equation S2. Theoretical capacity of NWO with W^{6+} valence

$$\mathrm{C(mAh}.g^{-1}) = \frac{n_e F}{3.6.\overline{M}}$$

$$C = \frac{\left(0.5e_{Na^+} + 1e_{W^{6+}\rightarrow 4^+} + 0.5e_{Nb^{5+}\rightarrow 4^+}\right).96{,}485}{3.6\ .\ 197.87}$$

$$C = 270.9mAh.g^{-1}$$

Figure S3. Electrochemical activation process of mixed Nb-W oxide (NWO) in 1 M NaOH at 50 mV s⁻¹ in cycles 2, 5, 10 and 13: (a) full cyclic voltammograms; (b) magnification of the anodic region; (c) magnification of the cathodic region.

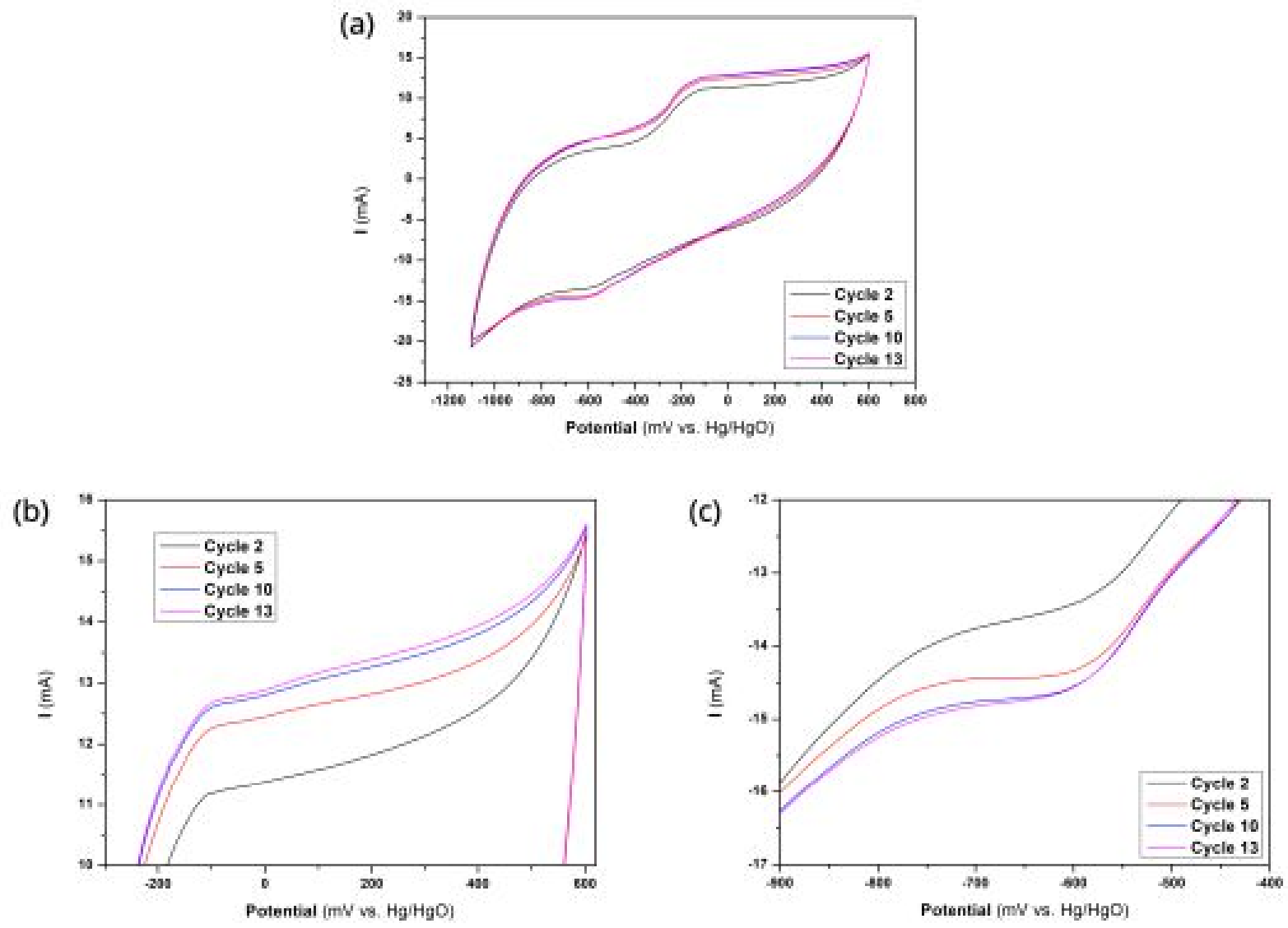

Supplement: Supplementary file 1 [file ao6c02992_si_001.pdf]
